# Ultrastructural visualization of trans-ciliary rhodopsin cargoes in mammalian rods

**DOI:** 10.1186/s13630-015-0013-1

**Published:** 2015-02-08

**Authors:** Jen-Zen Chuang, Ya-Chu Hsu, Ching-Hwa Sung

**Affiliations:** Department of Ophthalmology, Dyson Vision Research Institute, Weill Medical College of Cornell University, New York, NY 10065 USA; Department of Cell and Developmental Biology, Weill Medical College of Cornell University, New York, NY 10067 USA; The Margaret M. Dyson Vision Research Institute, Weill Medical College of Cornell University, 1300 York Avenue, LC313, New York, NY 10065 USA

## Abstract

**Background:**

Cilia are vital to various cellular and sensory functions. The pathway by which ciliary membrane proteins translocate through the transition zone is not well understood. Direct morphological characterization of ciliary cargoes in transit remains lacking. In the vertebrate photoreceptor, rhodopsin is synthesized and transported from the inner segment to the disc membranes of the outer segment (OS), which is a modified cilium. To date, the membrane topology of the basal OS and the mechanisms by which rhodopsin is transported through the transition zone (i.e., connecting cilium) and by which nascent disc membranes are formed remain controversial.

**Results:**

Using an antibody recognizing its cytoplasmic C-terminus, we localize rhodopsin on both the plasma membrane and lumen of the connecting cilium by immuno-electron microscopy (EM). We also use transmission EM to visualize the electron-dense enzymatic products derived from the rhodopsin-horseradish peroxidase (HRP) fusion in transfected rodent rods. In the connecting cilium, rhodopsin is not only expressed in the plasma membrane but also in the lumen on two types of membranous carriers, long smooth tubules and small, coated, filament-bound vesicles. Additionally, membrane-bound rhodopsin carriers are also found in close proximity to the nascent discs at the basal OS axoneme and in the distal inner segment. This topology-indicative HRP-rhodopsin reporter shows that the nascent basalmost discs and the mature discs have the same membrane topology, with no indication of evagination or invagination from the basal OS plasma membranes. Serial block face and focus ion beam scanning EM analyses both indicate that the transport carriers enter the connecting cilium lumen from either the basal body lumen or cytoplasmic space between the axonemal microtubules and the ciliary plasma membrane.

**Conclusions:**

Our results suggest the existence of multiple ciliary gate entry pathways in rod photoreceptors. Rhodopsin is likely transported across the connecting cilium on the plasma membrane and through the lumens on two types of tubulovesicular carriers produced in the inner segment. Our findings agree with a previous model that rhodopsin carriers derived from the cell body may fuse directly onto nascent discs as they grow and mature.

**Electronic supplementary material:**

The online version of this article (doi:10.1186/s13630-015-0013-1) contains supplementary material, which is available to authorized users.

## Background

The cilium is a vital organelle harboring receptors and channels for a variety of sensory functions. Human mutations of genes important for ciliary structure and/or protein trafficking have been linked with ciliopathic diseases. The pathway by which ciliary membrane proteins translocate from the cell body through the proximal diffusion barrier, known as the “transition zone (TZ),” remains to be elucidated. Prior studies have used combinations of biochemistry, reverse genetics, and functional analyses, but morphological delineation of ciliary membrane cargoes during TZ transit has been largely lacking.

**Figure 1:**
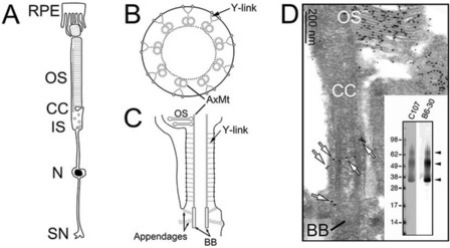
**Architectures of rod and CC and rhodopsin immuno-EM. (A)** Schematic diagram of a mammalian rod showing its cellular compartments: OS, CC, inner segment (IS), nucleus (N), and synapse (SN). The distal tip of the OS directly contacts the retinal pigment epithelial (RPE) cells that phagocytose the shed discs as part of OS renewal. **(B, C)** Schematic diagrams of cross-sectional **(B)** and longitudinal **(C)** views of the CC. *BB* basal body. **(D)** An immuno-EM electron micrograph demonstrates the distribution of rhodopsin (detected by C107 Ab followed by 10-nm gold-conjugated secondary Ab). White arrows point to rhodopsin in the CC. Insert: immunoblots of bovine rod OS lysates probed with either anti-rhodopsin C-terminus rabbit Ab C107 or anti-rhodopsin N-terminus monoclonal Ab B6-30. Arrowheads point to monomers, dimers, and higher-order oligomers of rhodopsin.

The photoreceptor is a ciliated sensory neuron that provides an attractive model system to study trans-ciliary transport for several reasons. (i) Mammalian photoreceptors use a large modified, non-motile cilium called the outer segment (OS) to sense and transduce light. OSs are packed at high density in the outer retina in a parallel orientation, allowing convenient access to large numbers of samples. (ii) Each rodent OS (~1.4 μm in diameter; ~17-28 μm in length) contains several hundred disc membranes that host 10–100 million rhodopsin photopigment molecules (Figure 1A; reviewed in [1,2]). OS disc membranes undergo constant renewal [3]. At the distal end of the OS, aged discs are shed and phagocytosed by the neighboring retinal pigment epithelial cells, while at the proximal end of the OS nascent discs are formed by the incorporation of rhodopsin (and other OS proteins and lipids) synthesized in the part of the cell body called the inner segment. Thus, the transport of rhodopsin from the inner segment to the OS through the connecting cilium (CC) is highly active and, hence, favorable for ciliary transport studies. (iii) Several lines of evidence suggest that the CC is the ciliary TZ of the photoreceptor. First, almost all of the TZ complex components found in simple cilia are localized to the CC [4,5]. Second, the CC contains Y-links, a signature TZ structure composed of a large protein complex that connects the 9 + 0 axonemal microtubules (AxMT) to the overlying plasma membrane (PM) (Figure 1B; [6]). Freeze fracture analysis showed that the ciliary necklace (perhaps representing the intramembranous particles constituted by the outer ends of the Y-link) is distributed throughout the entire CC PM [5,7]. It is generally thought that the Y-link functions as a diffusion barrier to regulate the travel of molecules in and out of the cilium [6]. The CC is ~10 times longer than the TZ of a typical cilium and thus provides higher spatial resolution for the ultrastructural characterization of ciliary cargo in transit.

**Figure 2:**
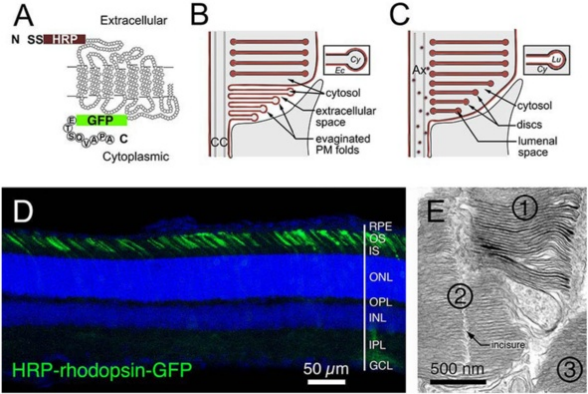
**Schematic diagrams of HRP-rhodopsin reporter, its expected expression in different disc genesis models, and its expression pattern in transfected rods. (A)** A diagram of the HRP-rhodopsin reporter in the context of its transmembrane helices. The N-terminus is tagged with HRP and the signal sequence (SS) of Drosophila *wingless* gene, while the C-terminus is tagged with GFP and the rhodopsin 1D4 epitope. **(B)** A schematic diagram delineating the predicted distribution of DAB precipitates (burgundy) in the scenario that the OS base has evaginated PM protruding into the extracellular (Ec) space. Inset depicts the DAB deposits that would be expected on the convex side of evaginated PM outfolds that are filled by cytosol (Cy; gray). **(C)** A schematic diagram delineating the predicted distribution of DAB precipitates (burgundy) in the scenario that the base of the OS is completely enveloped by the PM and surrounded by cytosol. DAB would also be observed on the luminal sides of rhodopsin-bearing vesicles and on the luminal (Lu) side of disc membranes (inset). **(D)** A representative confocal image of a retinal slice containing HRP-rhodopsin transfected rods. **(E)** An electron micrograph revealing the OS of three rods after DAB reactions. Cell No. 1 is positive, whereas No. 2 and No. 3 are negative for HRP activity. An arrow points to the disc incisures of cell No. 2.

Despite these advantages, the mechanisms underlying the ciliary transport of rhodopsin and disc genesis during OS renewal, two interrelated processes, are still not well understood. This is, in part, due to controversy over the proximal OS membrane topology [1,2,8]. Steinberg et al. [9], using transmission electron microscopy (TEM), observed 5–15 gradually elongated, ciliary PM outfolds situated at the distal CC (Figure 2B). They hypothesized that nascent discs are formed one at a time through a poorly understood “sealing” process (fusion of the most distal outfold with the basal OS PM). While this theory is often referred to as the “open disc model,” the outfolds are actually evaginated PM (i.e., filled with cytosol and surrounded by extracellular space; inset in Figure 2B), rather than disc membranes. Note that disc membranes had enclosed lumens and were surrounded by cytosol. Based on rapid freezing/freeze-etching EM images, Obata and Usukura [10,11] proposed a second model, the invagination model, in which they hypothesized that nascent discs are formed by fusing vesicles pinocytosed from the basal OS PM. Both the evagination and invagination models are compatible with a “patency” phenomenon [12,13]. That is, extracellularly applied fluorescent dyes appear within the lumens of nascent discs, presumably by entering during the “disc sealing” process. Nonetheless, the patency phenomenon has only been demonstrated in rods of lower vertebrates, but not in several mammalian species tested [13].

The TEM images of rodent rods obtained in our lab did not show evidence of outwardly folded PM between the CC and the OS [14]. The few basalmost nascent discs/cisternae are smaller than the mature discs (Figure 2C), morphologically resembling the evaginated PM described by Steinberg et al. [9] and others [15,16], except that they are enveloped by the OS PM. More recently, using cryo-EM, a method without chemical fixation, Gilliam et al. [17] showed that the base of the rod OS is completely “closed” by the PM as well. Due to the low tissue penetration of cryo-EM method, dissociated rod axonemes were used, so concerns remain whether the extensive axoneme isolation procedures may have had an impact on the OS membrane structure.

Primarily based on rhodopsin immunolocalization studies [1,8,18-24], several models have been proposed to explain the transport route of rhodopsin from the inner segment to the OS. The current predominant model proposes that rhodopsin translocates through the CC along the ciliary PM via intraflagellar-mediated transport (IFT) [25-28]. In agreement with this model, post-embedding immuno-EM detected rhodopsin on the CC PM [23,29,30]. Furthermore, transgenic mice constitutively null for KIF3A (a key motor that drives IFT) [28] and hypomorphic for IFT88 (a key IFT component) [27] had rhodopsin mislocalized from the OS. Later studies using conditional knockout mice in which *kif3A* was deleted after OS maturation, however, suggested that KIF3A is dispensable for the OS localization of rhodopsin [31]. Thus, whether or not the IFT pathway is critically involved in rhodopsin ciliary transport remains unclear.

Vesicular structures have been previously seen in the CC lumen of both developing and adult rods through the use of the rapid freeze/etch, TEM, and cryo-EM methods [10,14,17]. Additionally, several disease mouse models exhibit membrane-bound vesicles abnormally accumulated at the base of the rod CC, in the CC lumens, the basal OS axonemes, and/or the extracellular space near the CC; this phenotype has often been associated with rhodopsin mislocalization [14,17,32,33]. While indicative, direct evidence demonstrating that the CC vesicles are the carriers for rhodopsin remains lacking.

Previous studies showed that horseradish peroxidase (HRP), when being expressed at the luminal side of a membrane protein, can be used as a genetic tag for ultrastructural localization study based on the electron-dense enzymatic products derived from HRP activity [34-36]. HRP expression causes little or no effect on membrane integrity, and its relatively uniform labeling allows identification of small intracellular structures, such as synaptic vesicles [36]. Unlike many antigen epitopes, HRP activity is less affected by microenvironmental pH and glutaraldehyde fixation [37]. Thus, HRP can be visualized in hard-fixed samples with better-preserved ultrastructural morphology. Furthermore, the HRP substrates, H_2_O_2_ and 3,3′-diaminobenzidine (DAB) tetrahydrochloride hydrate, are far smaller than a nano-gold-conjugated antibody (Ab) and therefore have better tissue penetration. This is especially important when detergent is omitted during the labeling procedure to better preserve membrane ultrastructure.

In this paper, we have combined immuno-EM, TEM, and three-dimensional scanning EM (3D-SEM) approaches to comprehensively delineate the nature of rhodopsin-bearing transport carriers and their spatial relationship to the CC, as well as the membrane topology of the basal OS. These findings greatly improve our understanding of disc genesis and rhodopsin’s OS transport pathways.

## Methods

### Reagents and animals

A cDNA fragment encoding a signal sequence fused to HRP was PCR amplified from the ssHRP-TM vector [36] and inserted into the N-terminus of rhodopsin-GFP-1D4 plasmid (1D4 is an epitope encoded by ETSQVAPA) [38]. The addition of the signal sequence and resulting glycosylation of HRP are necessary for its enzymatic activity [36]. The entire coding sequence of ssHRP-rhodopsin-GFP-1D4 plasmid was then transferred to the pCAG vector (gift of Connie Cepko [39]) for electroporation. All chemicals were purchased from Sigma unless otherwise mentioned. Rabbit anti-rhodopsin C-terminus Ab C107 was generated using maltose-binding protein fusion containing rhodopsin’s C-terminal 39 residues as an antigen (Cocalico Biochemicals, PA). A 10-nm gold-conjugated anti-rabbit IgG (Electron Microscopy Sciences) was also used. Isolation of bovine rod OS [40] and Odyssey-based immunoblotting assay were carried out using standard protocols.

All methods that involved live animals were approved by the Weill Medical College of Cornell University Institutional Animal Care and Use Committee.

### Immuno-EM

For post-embedding immuno-EM, CD1 mouse retinas were harvested by transcardial perfusion with 20 mL of 4% paraformaldehyde plus 0.1% glutaraldehyde. Retina blocks were quenched in 0.1 M glycine in phosphate-buffered saline (PBS) for 5 min, rinsed with PBS, and followed by gradient dehydration in ethanol solutions. Retina blocks were then embedded in LR-White resin (Electron Microscopy Sciences) and polymerized for 48 h at 50°C, following the manufacturer’s instructions. Ultrathin sections (70 nm) were cut and collected on nickel grids. The grids were then baked at 60°C for 1 h. Prior to immunolabeling, grids were etched with 1% H_2_O_2_ and rinsed in deionized water. Grids were blocked by 1% bovine serum albumin (BSA)/PBS and then incubated with primary Abs at room temperature overnight. Grids were washed in PBS and incubated with 10-nm gold-conjugated secondary Abs (Electron Microscopy Sciences) in PBS containing 1% BSA/fish gelatin (Amersham Biosciences) at room temperature. After final washes in PBS and then water, grids were counter stained with uranyl acetate and lead citrate for final examination on a Philips CM10 electron microscope. Approximately 150 rods were examined on the scope, and electron micrographs of a total of 52 rods were taken for detailed analysis.

### Retinal transfection, light microscopy examination, HRP activity detection, and TEM

For rod transfection, 2 μg of HRP-rhodopsin reporter plasmid was injected subretinally, followed by electroporation of the eyes of postnatal day 0 Sprague-Dawley rats, as described [39,41]. Animals were housed in 12-h light-dark cycles up to postnatal day 21 (at which time the rods had reached maturation) and harvested around noon under normal room light using cardiac perfusion, as previously described [14]. Retinal pieces (~2 × 4 mm) isolated from fixed eyecups were embedded in 5% low-melting agarose and cut by vibratome (40 μm thick). Green fluorescence in transfected rods was directly observed by Leica TCS SP2 spectral confocal microscopy.

For HPR detection, freshly cut, fixed retina vibrotome sections were quenched in 50 mM NH_4_Cl/0.1 M phosphate buffer, pH 7.4 (PB) for 10 min or 1% NaBH4/PB for 30 min at room temperature. After washes in Tris-buffered saline, sections were treated with 0.22 mg/mL DAB in Tris-buffered saline plus 1.5% H_2_O_2_ and monitored under a microscope for proper development of chromogenic products, typically 4–12 min. The reaction time was chosen with the aim of developing the DAB precipitates for a good signal-to-noise contrast, rather than the highest labeling density. Reactions were stopped by transferring sections to fresh Tris-buffered saline and then to PB. Sections were then subjected to EM analysis following routine procedures [14]. Briefly, the labeled vibrotome sections were fixed by 2% osmium tetroxide in PB, dehydrated with graded ethanol, embedded in Epon, and then subjected to ultrathin sectioning. The ultrathin sections were then treated with uranyl acetate-lead citrate counterstaining before examination under a Philips CM10 microscope.

### Serial block face scanning electron microscopy

Adult CD1 mice were transcardially perfused with a mixture of 2.5% glutaraldehyde and 4% paraformaldehyde in 0.1 M cacodylate buffer, pH 7.4. The eyes were then enucleated and post-fixed for an additional 2 days on ice in the same solution. Small pieces of eyecup (~2 × ~2 mm) were processed for *en bloc* fixation and staining, as described [42,43]. Briefly, specimens were incubated with 1.5% potassium ferrocyanide, 2 mM calcium chloride, and 2% osmium tetraoxide in 0.15 M cacodylate buffer, pH 7.4 for 1 h on ice followed by treatment with thiocarbohydrazide solution for 20 min at room temperature then with 2% osmium tetroxide fixation for 30 min at room temperature. After fixation, the tissue block was incubated in 1% uranyl acetate at 4°C overnight. The following day, *en bloc* Walton’s lead aspartate staining was performed at 60°C for 30 min, followed by serial ethanol dehydration and embedding in Embed-812 (Electron Microscopy Sciences). Tissue blocks were mounted and electrically grounded with the application of silver paint. The surface of the specimen was sputter coated with a thin layer of gold/palladium and subjected to serial block face imaging using a 3View ultramicrotome. Samples were imaged every 65 nm at a resolution of ~3.5 nm per raw pixel.

### Focused ion beam scanning electron microscopy

Retinal tissue blocks of adult C57BL/6J mice were prepared and *en bloc* stained as described above. Block face images of the samples that were precision milled every 10 nm (with a pixel resolution of ~5 nm/pixel in *x*, *y*, and *z*) or 5 nm (with a pixel resolution of ~3.5 nm/pixel in *x*, *y*, and *z*) were collected by FEI Helios NanoLab 650 microscope. Images were processed using Serial Sections Alignment Programs of IMOD/eTomo to correct drifting caused by the 30° angle from the block face during imaging.

## Results and discussion

### Immuno-EM of rhodopsin in mouse rod CC

Immunolabeling patterns can be influenced by several factors (e.g., availability of the antigen epitopes, fixation/embedding/permealization conditions). Previous studies by Hicks and Molday [44] found that, under the same labeling conditions, rhodopsin was predominantly labeled on the plasma membranes by an Ab against the extracellular N-terminus but predominantly labeled on the disc membranes by an Ab against the cytoplasmic C-terminus. They explained the difference as due, in part, to the accessibility of the antigen epitope.

The CC immunolabeling pattern of rhodopsin could also be influenced by the Ab and/or the conditions used. A previous study demonstrating the CC PM location of rhodopsin was carried out using a monoclonal Ab recognizing its N-terminus (facing the extracellular side) [23]. We decided to revisit the CC location of rhodopsin by performing immuno-EM of rhodopsin in mouse rods using a polyclonal Ab specifically recognizing rhodopsin’s C-terminus (Figure 1D; also see “Methods”). As predicted [44], the OS discs were heavily labeled by rhodopsin immunogolds. Rhodopsin immunogolds were also detected in the distal inner segments near basal bodies as well as in the CC (white arrows, Figure 1D). In the CC, rhodopsin immunogolds were localized to both the PM and the lumen in a roughly 1:2 ratio (*n* = 52). These labelings were immunospecific; they were absent when the primary Ab was omitted or when the primary Ab was presorbed by the antigen (not shown).

While these studies clearly indicated that rhodopsin is expressed in the lumen of the CC, the limited membrane preservation offered by the immuno-EM prevented us from characterizing the types of the transport carriers nor the detailed location of the rhodopsin distributed within the CC lumen.

### Characterization of the membrane topology of the OS base

Expression studies of ectopically expressed reporter molecules have been recognized as a useful means to complement the immunolabeling of endogenous molecules and to circumvent the technical problems associated with immunolabeling. Previous studies have shown that rhodopsin reporters transfected in rodents rods are predominantly localized to the OS [14,41]. Several retinitis pigmentosa mutant rhodopsins share a similar mislocalization pattern in transfected rat rods and conventional transgenic mouse rods [41]. These results indicate that transfected rhodopsin follows the same transport pathway used by endogenous rhodopsin. Thus, we set out to visualize the ultrastructural distribution of HRP-tagged rhodopsin reporter in transfected rods *in situ* based on electron-dense DAB reaction products. Since we tagged the HRP onto the N-terminus of rhodopsin (Figure 2A), the DAB precipitates are expected on the extracellular side of the PM, the luminal side of the disc membrane, and the luminal side of rhodopsin transport carriers. We reasoned that the precise distribution of the DAB deposits, as well as their interrelationship with the surrounding cytosol, could unambiguously demonstrate the membrane topology of the basal OS membranes (Figure 2B vs. 2C).

**Figure 3:**
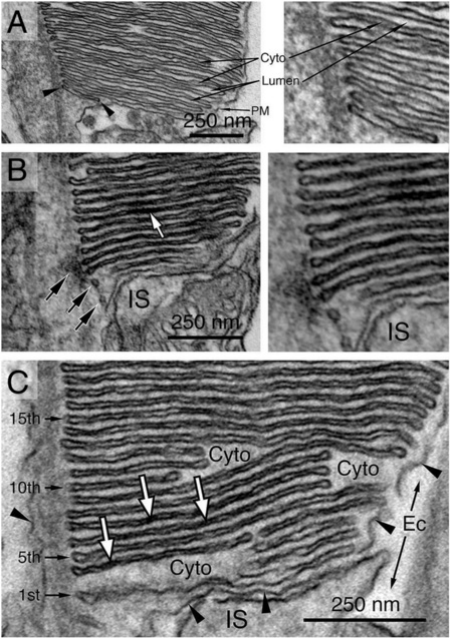
**Membrane organization of the basal OS.** Electron micrographs of the basal OS in unlabeled, non-transfected **(A)** and DAB-labeled, transfected **(B, C)** rods. Arrowheads in **(A)** point to the nascent discs/cisternae at the basal-most OS, which are surrounded by cytosol and enveloped by the OS PM. Arrows in B point to small tubulovesicles containing DAB precipitates inside their lumens; some of them look as if they are undergoing fusion with the adjacent discs. White arrows in **(B)** and **(C)** point to the intense DAB precipitate inside the lumens of the disc membranes. The bottom 1st to 15th discs (in **C**) are clearly surrounded by the OS cytosol (Cyto). Despite the occasional PM discontinuity (arrowheads in **C**), the differential electron density between the OS cytosol and extracellular (Ec) space clearly shows that the base of the OS is “closed” without evaginated PM. *IS* inner segment.

GFP and a 1D4 epitope (consisting of eight C-terminal residues of rhodopsin) were also added to the C-terminus of the HRP-rhodopsin reporter to facilitate the identification of transfected rods and ensure OS targeting [38,45], respectively (Figure 2A). We first confirmed the targeting specificity by showing that the green fluorescence derived from the transfected HRP-rhodopsin was predominantly localized to the OS (Figure 2D). We then subjected the transfected retinal vibrotome sections to DAB chromogenic reaction, followed by ultrathin sectioning and TEM. The transfected rods (e.g., cell 1 in Figure 2E) can be readily identified and distinguished from the neighboring non-transfected rods (e.g., cells 2 and 3 in Figure 2E) based on their more intense electron density. High magnification examination of untransfected rods showed that the OS cytosol was slightly darker than the disc lumen and the extracellular space (Figure 3A). By contrast, the DAB products in transfected rods were specifically deposited inside the disc lumen, giving it a much darker appearance than the cytosol (white arrows, Figure 3B, C).

In either non-transfected or transfected rods, the PM and the cytosol of the OS surrounded the entire disc stacks, including the most proximal cisternae (arrowheads, Figure 3A) and tubulovesicles (black arrows, Figure 3B). The previously described “open discs” at the basal OS with their extracellular surface labeled by DAB precipitates were not detected. No internalized membrane profiles indicative of invagination were observed either.

Others have considered the possibility that the extra PM artificially gained during the chemical fixation (or other experimental procedures) might somehow encompass the evaginated outfolds, rendering a “closed” appearance to the OS base [2,8]. If this were true, one would expect to see the DAB products on the convex sides of these “folds” (Figure 2B), regardless of whether or not they are enwrapped by the PM. That was however not the case. All of the basalmost discs had the DAB products expressed inside their lumens (Figure 3B, C).

### Ultrastructural characterization of the rod CC axoneme

**Figure 4:**
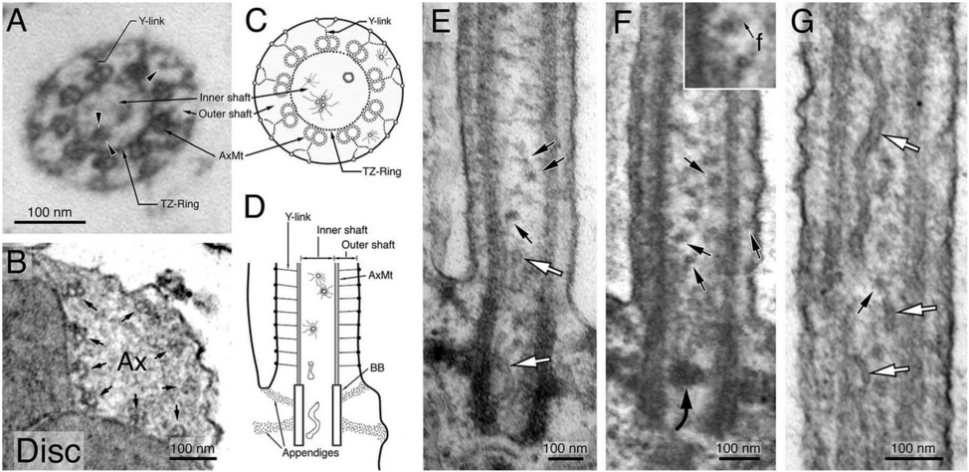
**Ultrastructural characterization of membrane structures within the CC and basal OS axoneme.** Morphological characterization of the CC and OS axonemes of untransfected, unlabeled Sprague-Dawley rat rods. **(A, B)** Electron micrographs of the representative cross-sectional view of the CC **(A)** and the basal OS axoneme **(B)**. Both Y-link and TZ-ring are prominent in the CC **(A)** but not in the OS axoneme **(B)**. Arrowheads in **(A)** point to small vesicles, present in both inner and outer shafts. Arrows in **(B)** point to the nine AxMt doublets. **(C)** and **(D)** are schematic diagrams depicting simplified versions of the electron micrographs of **(A)** and **(E)**, respectively. **(E–G)** Representative longitudinal sectional views of the CC. White arrows point to smooth membrane tubules; black arrows point to some of the representative coated small vesicles that often had filament **(f)** attached (an enlarged view is shown in the inset of **F**). A curved arrow points to a vesicle cluster that happens to be situated in the middle of a basal body.

To identify the putative rhodopsin carriers that transit through CC shafts, we first conducted a systematic morphological characterization of the CC axoneme in wild-type rat and mouse rods without any transfection or labeling. Consistent with previous freeze fracture analyses [7,46], our TEM images showed Y-link structures in almost all of the cross-sections cut through the entire rod CC (Figure 4A), but not in the basal OS axoneme (Figure 4B). These electron micrographs also reveal a prominent ring-like structure that connects the nine AxMT doublets, herein referred to as the “TZ-ring”. The TZ-ring was also a morphological feature specific to the CC region (Figure 4A); it was undetectable in the OS axoneme (Figure 4B). A similar ring structure has been previously described in the TZ of motile cilia in *Caenorhabditis elegans* [47].

**Figure 5:**
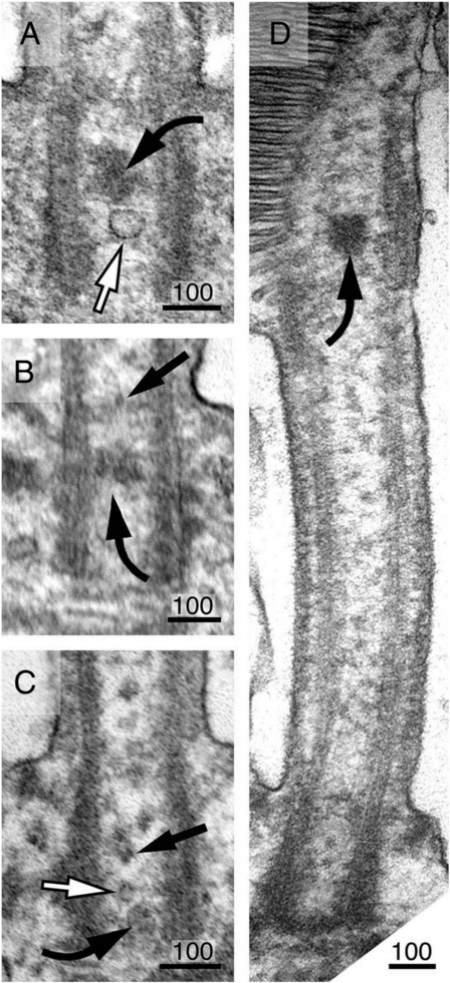
**Morphological characterization of CC vesicular structures.** Ultrastructural analysis of clustered CC vesicles in untransfected, unlabeled rods of Sprague-Dawley rats **(A, B, C)** and C57BL/6J mice **(D)**. Representative electron micrographs of four longitudinal sections of the CC or OS axonemes in unlabeled rods. Curved black arrows point to the vesicle clusters in between the basal bodies **(A, B, C)** or in the proximal OS axoneme **(D)**. While some of these vesicular clusters appear to be tightly associated with each other and have a compact, dark appearance **(A, D)**, others are more loosely associated and less electron-dense **(B, C)**. White arrows point to non-coated clear vesicles. Black arrows point to small coated vesicles. Scale bars are in nm.

The TZ-ring appeared to compartmentalize the CC lumen into an “inner” and an “outer” CC shaft (Figure 4A, C, and D). The estimated diameter of the inner shaft is ~125 nm, and the distance between the TZ-ring and the PM (i.e., outer shaft) is ~70 nm. Two main membranous structures were readily detected in the CC shafts. The first were membranous tubules; these tubules had smooth inner and outer surfaces with an outer diameter of 20–47 nm and variable length (white arrows, Figure 4E, G). These clear tubules were seen more frequently in the inner shafts than in the outer shafts. We also observed many vesicles with a clear surface (white arrows, Figure 5A, C); some of them conceivably represented obliquely sectioned views of the clear tubules.

The second type of structures had a particle appearance. However, close inspection showed that these structures were, in fact, small vesicles. These vesicles had heavy coats, and an estimated diameter was up to ~20 nm (arrowheads, Figure 4A; black arrows, Figure 4E–G). These vesicles were distributed either singly or tethered to each other by thin “tuft-like” filaments (Figure 4A, E–G). These vesicles were predominantly found in inner shafts (Figure 4A, E–G) and to a lesser degree in the outer shaft (Figure 4A, F). In some cases, the coated vesicles were tightly clustered, rendering a dark fuzzy ball-like structure (black curved arrows, Figures 4F and 5A–D). The vesicle clusters varied in both size (~60–100 nm in diameter) and shape and were found anywhere between the proximal CC to the basal OS axonemes (but rarely in the distal OS axoneme). The heterogeneous expression pattern of the dark fuzzy structures suggests they are not the counterpart of the amorphous electron-dense matrix structure seen in the basal body lumens of motile cilia [48,49].

### Morphological characterization of rhodopsin carriers

**Figure 6:**
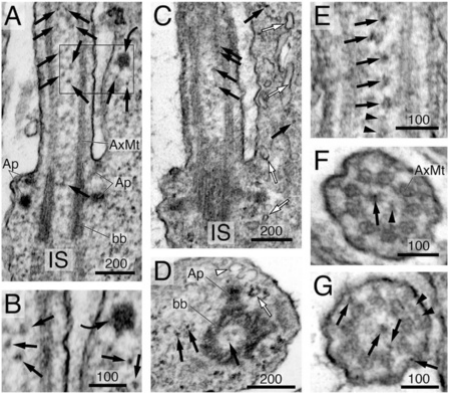
**Ultrastructural analysis of vesicular structures expressing HRP-rhodopsin.** Morphological characterization of rhodopsin-laden vesicles and tubules of HRP-rhodopsin transfected rods **(A–G)**. Representative longitudinal **(A–C, E)** and cross-sectional **(D, F, G)** views of rods are shown. **(B)** Enlarged view of the boxed area in **(A)**. Curved black arrows point to DAB-labeled vesicle clusters. Black arrows represent DAB-positive vesicles; black arrowheads represent DAB-negative vesicles; white arrows represent DAB-positive tubules; white arrowheads point to the DAB-negative tubules. For simplicity, not all examples are labeled by arrows or arrowheads. *Ap* appendage; *bb* basal body; *IS* inner segment. Scale bars in nm.

**Figure 7:**
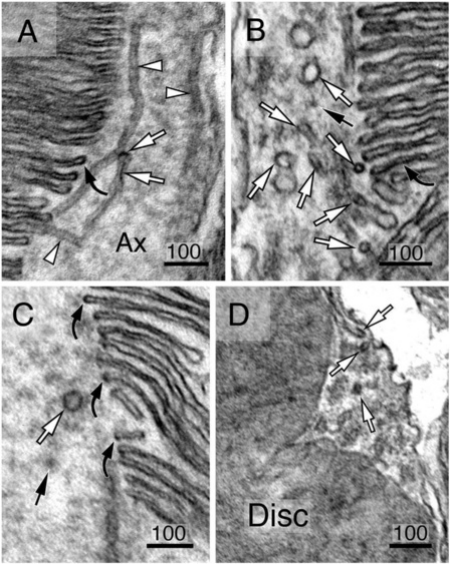
**Morphological characterization of HRP-rhodopsin-carrying vesicular tubules in the basal OS axonemes.** Electron micrographs of longitudinal **(A–C)** and cross-sectional **(D)** views of proximal OS axonemes of HRP-rhodopsin transfected rods showing the membrane tubules with (white arrows) or without (white arrowheads) dark DAB precipitates. The intensity of DAB labeling in the branches or distal tips of the tubules is comparable to that of adjacent discs (curved black arrows). The diameter of the distal ends of these tubules is also similar to that of the disc membrane. Black arrows point to DAB-labeled small vesicles. Scale bars in nm.

We subsequently performed an ultrastructural analysis of DAB-reacted, HRP-rhodopsin transfected rods to search for rhodopsin carriers. In most of the rods examined (~92%, *n* = 191 of longitudinal sections), we were able to detect small coated vesicles having their lumens filled with DAB precipitate, hence, darker compared to the unlabeled lumens (black arrows vs. black arrowheads, Figure 6A–G). In the CC inner shaft, the frequency of detected DAB-labeled vesicles was up to ~260 vesicles per μm^2^. The DAB-labeled vesicles were also seen in the outer shaft (black arrows, Figure 6G) and basal body lumens (Figure 6D). DAB-labeled membranous tubules were observed as well (white arrows, Figure 6C, D). Interestingly, in the basal OS axonemes, the tubulovesicular structures often had the strongest DAB labeling at their branches and/or tips, which are in close juxtaposition to the nascent discs, as if they were about to fuse (white arrows, Figure 7A–D).

Both the DAB-labeled coated vesicles, either singly or in clusters (black arrows or curved arrows, Figure 6A–D), and DAB-labeled tubules (white arrows, Figure 6C, D) were abundant in the apical region of the rod inner segment. These tubular vesicle structures shared similar morphological features to those found in CC lumens, indicating that rhodopsin carriers seen in the CC were generated in the inner segments.

Finally, the ciliary PM and the apical inner segment PM of almost all transfected rods also had positive DAB reactivity (Figures 3B, C and 6A–G), consistent with the notion that rhodopsin is expressed on the PM of the CC.

### Serial SEM examination of rod CC and membrane carriers

**Figure 8:**
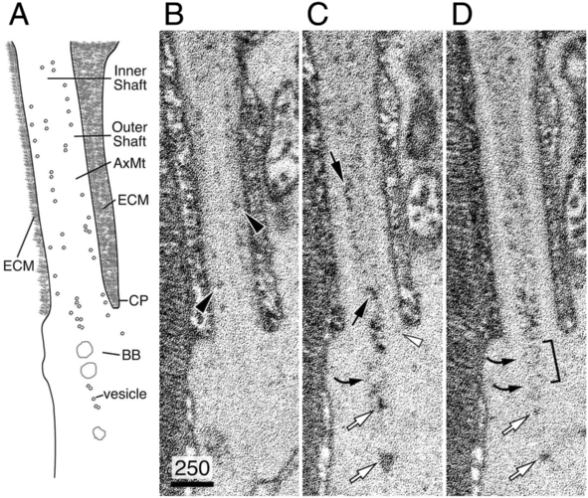
**SBF-SEM analysis of CC membrane carriers. (A)** A cartoon illustrating the results presented in **(B–D)**. The illustration includes the region from the distal inner segment to the CC in a mouse rod. Our staining/fixation protocol predominantly labels plasma membrane, intracellular membrane structures, and the extracellular matrices (ECM) in the CP, but non-membranous, cytoplasmic constituents, such as AxMT, basal body, and rootlets were not detected. **(B–D)** Three representative consecutive block face images collected by SFB-SEM (spanning 70 nm) revealed the vesicles located in the CC outer shaft (black arrowheads) and those in the CC inner shaft (black arrows). White arrowhead points to the vesicles at the CC outer shaft entry site. White arrows point to the vesicles towards the base of the CC as well as those aligned inside (or just beneath) the basal body lumens. Curved black arrows point to large clear vesicles. Bracket points to the predicted position of the basal body. Scale bar in nm.

**Figure 9:**
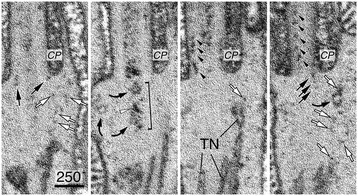
**SBF-SEM analysis of CC membrane carriers near CC entry sites.** Four representative consecutive block face images collected by SFB-SEM (spanning 70 nm). Black arrows point to membrane vesicles in or at the entry site of CC outer shaft. Black curved arrows point to large clear vesicles. Black arrowheads point to strings of vesicles inside the CC outer shaft. White arrows point to small vesicles closely associated with or at the vicinity of inner segment tubular network (TN). White arrowhead points to a vesicle cluster inside the basal body. Curved black arrows mark the large clear vesicles present in the basal body lumen (second panel) and distal inner segment (fourth panel). The predicted position of the basal body is labeled by a bracket. Scale bar in nm.

**Figure 10:**
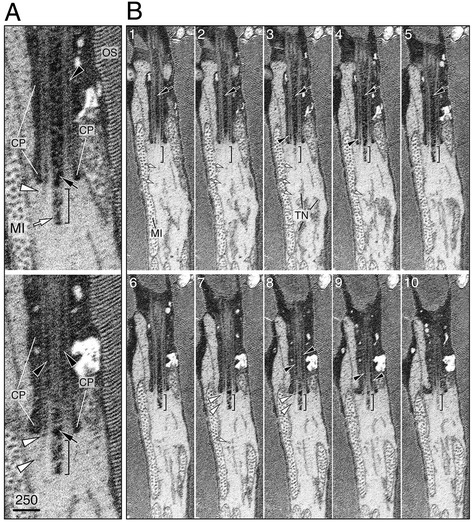
**FIM-SEM analysis of the spatial distribution of CC transport carriers. (A)** Two enlarged block face views of the rod OS and inner segment junction. Black arrows point to the membrane carriers in the inner shafts of the CC; white arrows point to the membrane carriers in the basal body lumens situated exactly beneath the CC inner shaft. Black arrowheads point to the membrane carriers in the outer shaft of the CC. White arrowheads point to a string of vesicles about to enter the outer shaft. The extracellular matrices in the CP are also labeled. **(B)** A series of ten block face views (labeled one through ten) taken from every other 10-nm section of Additional file 1: Movie 1. White arrows point to a vesicle string in the inner segment that appears to link to the vesicle stream in the CC inner shaft, some of these vesicles had a close spatial relationship with a membrane compartment formed by the elongated tubular network (TN) (frames 4, 7). White arrowheads point to a continuous string of vesicles at the CP base and CC outer shaft. Brackets mark the predicted basal body location. *MI* mitochondria. Scale bar in nm.

State of the art 3D-SEM techniques have recently been introduced to image large tissue volumes, including retinas [50]. There are two 3D-SEM methods, FIM-SEM (Focused ion beam SEM) [51] and SBF-SEM (Serial block face SEM) [52], both involving the automatic acquisition and perfect alignment of consecutive block face sections from well-preserved specimens; for SBF-SEM, the sections are cut by ultramicrotome (70-nm slice *z* intervals), and for FIM-SEM, the sections are shaved by ion beam milling (5-nm or 10-nm slice *z* intervals). In order to visualize the membranous transport carriers, we took advantage of an *en bloc* post-fixation/staining protocol that greatly enhanced the preservation and the contrast of lipid-containing structures (e.g., plasma membrane, membranous organelles, and vesicles), but not the proteinaceous constituents [42,43,53,54]. This protocol also stained the highly glycosylated structures (e.g., glycocalyx) though. Our imaging showed that while the ultrastructural details of OS disc membranes as well as the membranes of the mitochondria in the inner segments were extremely well preserved, other intracellular structures including the AxMT, basal body, appendages, cytoplasmic microtubules, and rootlet were undetectable. Perhaps due to the presence of vesicles (see Figure 3A) and heavily glycosylated molecules [55,56] localized in the interphotoreceptor matrices, the extracellular space in the ciliary pockets (CP) was also darkly stained (Figures 8, 9, and 10).

**Figure 11:**
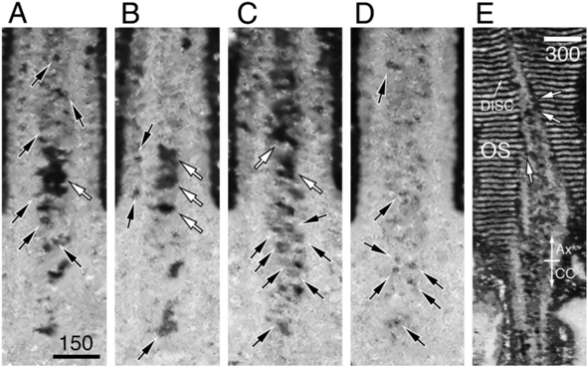
**FIM-SEM imaging of CC membranous carriers. (A–D)** Four representative block face FIM-SEM images reveal the membranous structures contained within the rod CC. Note that the density of the vesicle tubules was variable in different sectional views. In the CC sections that are less crowded, individual vesicles with clear membrane profiles were readily detectable in both inner and outer shafts (black arrows). White arrows point to the vesicle clusters of various sizes. Scale bar = 150 nm **(E)** A block face FIM-SEM image shows that the membrane structures sharing similar morphological features to those expressed in the CC are also abundantly expressed in the basal rod OS axoneme (Ax). Some of these vesicular membranes were immediately adjacent to the disc membranes as if they are about to fuse (white arrows point to examples). Scale bar = 300 nm.

**Figure 12:**
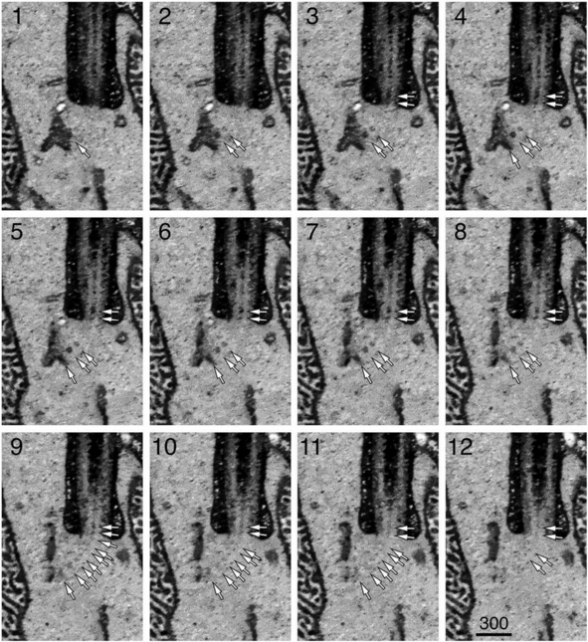
**Serial FIM-SEM imaging membrane vesicles at the CC entry sites.** Twelve consecutive images (collected at 5-nm *z* intervals) of basal rod CC. A string of vesicles can be connected from its inner segment location to its CC luminal location (white arrows). Scale bar in nm.

**Figure 13:**
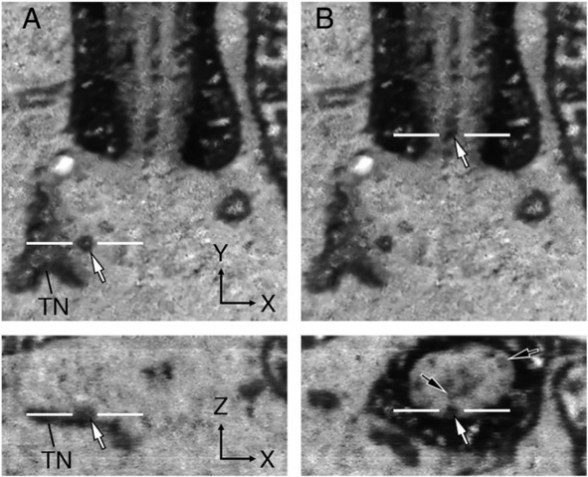
**Spatial relationship between the vesicles of upper inner segment and CC lumen. (A)** Enlarged views of Figure 12 (panel 4) showing both the block face *x*-*y* view (top panel) and orthogonal *x*-*z* view (bottom panel). The close spatial relationship between a vesicle (white arrow) and a membrane organelle in the upper IS is particularly prominent in the orthogonal view. **(B)** Top panel shows the same block face image of **(A)**; a white arrow points to a CC luminal vesicle that appears to be localized in the outer shaft based on the orthogonal view (bottom panel). Two black arrows label two out of several other CC luminal vesicles, which can be readily distinguished from the dark labeling of the ciliary PM and CP space.

We performed both SBF-SEM (Figures 8 and 9) and FIM-SEM (Figures 10, 11, 12, and 13, Additional file 1: Movie 1) to reconstruct the block face views of the rod OS and inner segment junctions. Despite the difference in sample cutting and imaging acquisition, the results obtained from these two methods consistently detected the membranous carriers in the inner shafts (black arrows, Figures 8, 9, and 10) and outer shafts (black arrowheads, Figures 8, 9, and 10) of CC lumens. The CC lumens containing a high density of membranous carriers were darkly stained and appeared black (Figures 10 and 12). In the CC lumens that had less densely packed membrane carriers, the profiles of individual vesicles (black arrows, Figures 11A–D and 13) and vesicle clusters (white arrows, Figure 11A–C) can be spatially resolved and easily detected. Both large (curved arrows, Figures 8 and 9) and small vesicles were readily discernible as well. The CC vesicles formed a continuous flow extending into the basal OS axonemal cytoplasm, where the dark vesicles were also abundant (Figure 11E). Some of these vesicles (white arrows, Figure 11E) were closely apposed to the disc membranes, which exhibited comparable electron density.

In almost all rods analyzed (*n* = 30), strings of vesicles were detected at the same site where the basal body lumen was localized (brackets in Figures 8, 9, and 10, Additional file 1: Movie 1) or exactly beneath that (white arrows in Figures 8, 9, and 10, black arrows in Additional file 1: Movie 1). These vesicles appeared to be part of a continuous flow of the vesicles in the CC inner shafts. Additionally, strings of vesicles were also seen near the base of the CP before they were channeled into the CC outer shafts (white arrowheads in Figures 8 and 10, white arrows in Figure 12, black arrows of Figure 9, and black arrowheads Additional file 1: Movie 1). In fact, CC vesicle strings were often connected to the vesicle strings localized in the upper inner segment (white arrows in Figures 10B and 12, black arrows in Additional file 1: Movie 1). Both *en face* (Figures 9, 10, and 13, white arrows in Additional file 1: Movie 1) and orthogonal (white arrows, Figure 13A) examinations showed some of these vesicles in the inner segments were immediately juxtaposed to a prominent organelle, which formed an extended tubular network spanning a long distance over the upper inner segment. These organelles were unlikely to be the *trans*-Golgi network, which is largely confined to the base of the inner segment [40]. We were tempted to speculate that some of the CC vesicles were emanating from these as-yet unidentified membranous organelles.

## Conclusions

The present study suggests that rhodopsin is translocated through the rod ciliary transition zone CC through multiple pathways, either on the PM or on membrane-bound carriers in the lumens. The membrane carriers, either large smooth vesicular tubules or small coated vesicles, are first synthesized in the inner segment, recruited at the base of the basal body, and then enter the inner shaft of the CC through the basal body lumen. Alternatively, they enter the CC outer shaft through the narrow cytoplasmic space between the AxMT/transition fibers and the ciliary PM.

### Multiple types of rhodopsin transport carriers

While the previous cryo-EM report identified membrane carriers in the outer shaft, only large protein particles (~45–110 nm) were detected in the inner shaft of the CC [17]. The conclusion was drawn based on the tomography analysis, which indicated that these large particles in the inner shaft had a different density profile compared to a typical phospholipid vesicle. On the other hand, several independent approaches in this paper all suggest the presence of CC membrane carriers in both the inner and outer shafts. First, our morphological characterization using high-resolution TEM reveals the presence of vesicles and tubules in the CC lumen. Second, our SEM analyses using a specific *en bloc* fixation/staining protocol reveals that the structures in CC lumens are membranous structures, not simple proteinous elements. The CC lumen vesicle density in the SEM images is evidently higher than that seen in the TEM images. We surmise it is due to the superiority of membrane preservation and membrane contrast due to the combinatory use of a stronger primary fixative and an *en bloc* post-fixation/staining protocol. Finally, we demonstrate the presence of integral membrane protein rhodopsin on both types of vesicular carriers in the CC lumen. The evidence includes (1) the immuno-EM reveals the expression of endogenous rhodopsin in the CC lumen and (2) DAB reaction products are specifically localized in the lumen side of the CC carriers in rods transfected with the topology-indicative HRP-rhodopsin reporter.

Our HRP-rhodopsin expression studies also reveal the expression of rhodopsin on the ciliary PM, in agreement with previous immuno-EM studies [23]. Although the HRP-rhodopsin method has better accessibility than immuno-EM, we emphasize that no detergent was used in our experiments, in order to better preserve the membranes. So the HRP expressed inside the CC lumen is less accessible to the DAB substrates than that expressed on the outer surface. By the same token, we would like to reiterate that any given labeling protocol can only reveal a partial view of total protein expression. Despite using an anti-rhodopsin N-terminus Ab, Wolfrum and Schmit [23] showed only CC plasma membrane labeling, while our protocol can readily detect the rhodopsin inside the CC lumen using an anti-rhodopsin C-terminus Ab. The relatively low density of rhodopsin labeled in the CC lumen is expected, likely due to technical reasons, including Ab competition with the high concentration of rhodopsin in the OS and poor Ab penetration due to the dense extracellular matrix/large protein complex coating on the ciliary membranes. Note that for light microscopic examination, CC proteins have typically been labeled using unfixed or mildly fixed retinas [26,57,58], which are not suitable for ultrastructural analysis. Thus, we caution that the ratio of rhodopsin labeling on the ciliary PM vs. lumens ([23], current study) should not be directly interpreted as the relative amount of rhodopsin transported on different pathways.

Despite the relatively narrower space and the presence of Y-links, to some surprise, both the previous cryo-EM [17] and present studies reveal the existence of membrane carriers in the CC outer shaft. This finding, nonetheless, dovetails with the previous observation that excess vesicles accumulate in both inner and outer shafts of the rods that have a disc fusion problem [14].

### Multiple trans-ciliary pathways in rod photoreceptors

While the biological meaning of having multiple types of rhodopsin carriers remains to be investigated, this finding suggests that various molecular motor systems might be involved in rhodopsin’s ciliary translocation. CC lumen-localized membrane carriers are within a close distance to the AxMT, making transport feasible through the use of the kinesin motor engaged on the AxMT (~25–30 nm; [59]). We speculate that the filamentous network linking the CC vesicles may be used to improve the processivity of motor-mediated transport activity and/or the efficiency of moving a large number of vesicles simultaneously. Filament-attached small vesicles have been seen in the developing mouse rod CC [10,60]. Obata et al. [10] showed that these filaments are myosin S1 fragment-decorated, short actin filaments; hence, actin-based motors (e.g., myosin VII [29,30]) may also be involved.

Coat formation is known to provide an effective means of concentrating membrane cargoes into patches; it is an evolutionarily ancient and conserved mechanism employed for selective sorting and preparing cargoes for transport from donor to recipient membranes [61]. The coat composition of the CC vesicle is currently unknown, a piece of information important to further delineate the mechanism underlying the ciliary targeting and TZ entry of rhodopsin. In this vein, it is interesting to note that Arl6-mediated recruitment of Bardet-Biedl syndrome (BBS) protein can induce coated patches on liposomes, a process important for the primary ciliary entry [62]. Mice with either the *bbs2* or *bbs4* gene deleted exhibited rhodopsin mislocalization [63-66], and *bbs4* knockout mice also had aberrant vesicle accumulation at the CC base [17].

Regardless of the nature of the transport mechanism(s), the existence of multiple transport pathways for rhodopsin implies the possibility that partial, subtle, or even undetectable rhodopsin mislocalization may happen when a single translocation pathway is suppressed.

### Rhodopsin’s disc incorporation and disc genesis

We imagine that the rhodopsin transported across the CC on the PM could diffuse into the OS PM, whereas that of transported on the membrane-bound carriers may directly fuse onto nascent discs. Supporting the latter notion, we found many rhodopsin-HRP carriers are in close proximity to the disc membranes, and the HRP labeling intensity of these carriers matches that of the disc membranes. Interestingly, some images show that rhodopsin is particularly concentrated on the edges of the membranous tubules in the basal OS axoneme (Figure 7A, B), indicating that the sorting continues even after these carriers have already entered the OS. We envision that the smaller vesicles with high rhodopsin density budded off from these tubules may be more fit for fusion to their immediately adjacent discs.

The above interpretation is in good agreement with the “vesicular targeting model” that we previously proposed for disc genesis [14]. This model was initially proposed based on the observations that (1) the membrane fusion protein syntaxin 3 and membrane tethering protein Smad anchor for receptor activation are enriched at the most basal OS, the area in which the fusion activity is presumably the most robust, and (2) perturbing either molecule in rodent rods caused aberrant disc formation as well as vesicular accumulation inside the CC and OS base. According to this model, multiple nascent discs can be formed simultaneously at the basal OS via fusion of membrane carriers transported from the inner segment; repeated fusions allow the nascent discs to grow to the size of mature discs. This also contrasts to the “open disc/disc rim formation” model which suggests that a single disc is formed at a time. The present study using the topology-indicative rhodopsin reporter shows that both the mature discs and the nascent discs share the same membrane topology, and therefore, all should be enveloped by the OS PM in rat rods. This argues against the presence of evaginated or invaginated PM at the OS base.

Except for one TEM study that showed sparse vesicles in the lumen of the primary cilium of chondrocytes [67], no vesicles have been localized to simple (primary) cilia. Our findings here suggest that vertebrate rods might have evolved from other ciliated cells and developed a specialized means to move ciliary cargo in bulk due to the high demands posed by disc formation.

## Additional file

Additional file 1:
**Movie 1.** FIM-SEM examination of the substructures of mouse rod CC and inner segment-OS junction. A movie presents a series of consecutive block face images collected by FIM-SEM (10-nm per frame, total 35 frames). Dark vesicles are highly abundant in both inner and outer shafts. Black arrowheads in frames 2–15 spotlight the trail of vesicles migrating towards the outer shaft of the CC; they appear in file as they prepare to enter the proximal end of the outer shaft (white arrowheads). In frames 16–26, a bended arrow marks the proximal end of the basal body, although the basal body *per se* is not visible. Many vesicles accumulate at the lumen of the basal body. In frames 17–35, black arrows point to the trail of vesicles moving towards the proximal end of the basal body for CC inner shaft entry. In frames 23–35, white arrows point to a membranous organelle that is formed by a network of interconnected tubular structures. Some of the vesicles have an appearance as if they were either passing through or budding from these tubular structures while en route to the base of the CC. Several still images of part of this movie are presented in Figure 10.

## Abbreviations

CC: Connecting cilium
OS: Outer segment
TZ: Transition zone
PM: Plasma membrane
EM: Electron microscopy
PBS: Phosphate-buffered saline
TEM: Transmission electron microscopy
SEM: Scanning electron microscopy
SBF-SEM: Serial block face scanning electron microscopy
FIM-SEM: Focused ion beam scanning electron microscopy
HRP: Horseradish peroxidase
AxMT: Axonemal microtubule
DAB: 3,3′-Diaminobenzidine tetrahydrochloride hydrate
BBS: Bardet-Biedl syndrome
CP: Ciliary pocket
